# Elastic–Plastic Deformation Analysis of Cantilever Beams with Tension–Compression Asymmetry of Materials

**DOI:** 10.3390/ma18245611

**Published:** 2025-12-14

**Authors:** Xiao-Ting He, Jing-Miao Yin, Zhi-Peng Chen, Jun-Yi Sun

**Affiliations:** 1 School of Civil Engineering, Chongqing University, Chongqing 400045, China; 202416021167t@stu.cqu.edu.cn (J.-M.Y.); chenzpcqu@163.com (Z.-P.C.); sunjunyi@cqu.edu.cn (J.-Y.S.); 2 State Key Laboratory of Safety and Resilience of Civil Engineering in Mountain Area, Chongqing 400045, China

**Keywords:** elastic–plastic analysis, cantilever beam, tension–compression asymmetry, elastic modulus, yield strength

## Abstract

In the elastic–plastic analysis of structures, the deformation problem of cantilever beams is a classical problem, in which it is usually assumed that the material constituting the beam has an identical elastic modulus and identical yield strength when it is tensioned and compressed. These characteristics are manifested graphically as the symmetry of tension and compression. In this work, we will give up the general assumption and consider that the material has the property of tension–compression asymmetry, that is, the material presents different moduli in tension and compression and different yield strengths in tension and compression. First, the elastic–plastic response of the cantilever beam with a concentrated force acting at the fixed end in the loading stage is theoretically analyzed. When the plastic hinge appears at the fixed end, the maximum deflection at the free end is derived, and in the unloading stage the residual deflection at the free end is also given. At the same time, the theoretical solution obtained is validated by the numerical simulation. The results indicate that when considering the tension–compression asymmetry of materials, the plastic zone length from the fixed end no longer keeps the classical value of 1/3 and will become bigger; the tension–compression asymmetry will enlarge the displacement during the elastic–plastic response; and the ultimate deflection in loading and the residual deflection in unloading are both greater than the counterparts in the classical problem. The research results provide a theoretical reference for the fine analysis and optimal design of cantilever beams.

## 1. Introduction

For most materials, it is generally assumed in the analysis that the mechanical properties in tension and compression are identical, that is, they are, from an objects or images perspective, “symmetric”; the term “tension–compression asymmetry” means that the materials have different mechanical properties in tension and compression [1,2,3]. The tension–compression asymmetry is a fundamental mechanical property of the material itself, which results in varying degrees of differences in elastic constant, yield strength, fatigue, and creep [4,5,6,7]. In structural analysis, if the constituent materials of structures possess such a property, then during the elastic–plastic response of the structure, the tension–compression asymmetry of materials will inevitably have a significant impact. For this purpose, this work will theoretically analyze the elastic–plastic deformation of a cantilever beam with tension–compression asymmetry for the first time.

The tension–compression asymmetry considered in this study means that not only in the elastic stage does the material has different elastic moduli in tension and compression (bimodular effect) [8], but also in the plastic yielding stage it has the strength differential effect (SD effect) [9,10]. For a long period of time, the study of the two effects has usually been clearly separated. It seems that the two are not related to each other. In fact, if one wants to track the entire elastic–plastic response, it is necessary to combine the two effects. Next, we will review the two effects of materials and their corresponding influences on structures.

The materials which present different moduli in tension and compression were firstly referred to as bimodular materials by Jones [11]. Bimodular materials include rubber [12], rock [13], polypropylene [14], and biological materials (for example, bone [15] and nacre [16]). For better understanding of the mechanical behavior of bimodular materials, researchers established two basic materials models. The first is Bert’s anisotropic materials model [17], which is established on the criterion of positive–negative signs in the longitudinal strain of fibers, and is widely used for the analysis of laminated composites, for example [18,19]. The second is Ambartsumyan’s isotropic materials model [20], established on the criterion of positive–negative signs of principal stresses. Therefore, we refer to the structure that takes into account the material’s bimodular effect as the bimodular structure. Presently, aimed at simpler structures, there were many analytical works concerning bimodular beams [21,22], bimodular plates [23,24], and bimodular shells [25,26], in which a simplified mechanical model on tension–compression subareas was used. For complex structures, however, we must adopt a finite element method with an iterative method [27,28,29]. At present, the study of bimodular structures has also been extended to bimodular truss, bimodular membrane, and tensegrity structures [30,31].

The strength difference effect (SD effect) of materials, reported in the literature over the past decades, is the difference between the axial stress of tension and compression. Many studies [32,33,34,35] have shown that non-ductile materials, including brittle metal materials such as cast iron, bronze, geological materials, and porous materials, will produce SD effects at yield, and SD effects are easily observed even for ductile materials such as steel. The study of the SD effect of materials seems to be a simple proposition, but it actually has a certain complexity. The reason is that the SD effect is generally related to yield criteria, including the earlier Tresca criterion, von Mises criterion, the subsequent twin-shear criterion [36], and the unified yield criterion [37]. In addition, the SD effect is related to the influence of hydrostatic stress on the mechanical behavior and deformation of materials [38]. The SD effect has been an interesting topic for a long time. Many works discussed some possible causes of SD effect, for example, different heat treatment conditions [39], micro-cracking in brittle structures, an influence of residual stresses, and the temperature [40].

**Figure 1:**
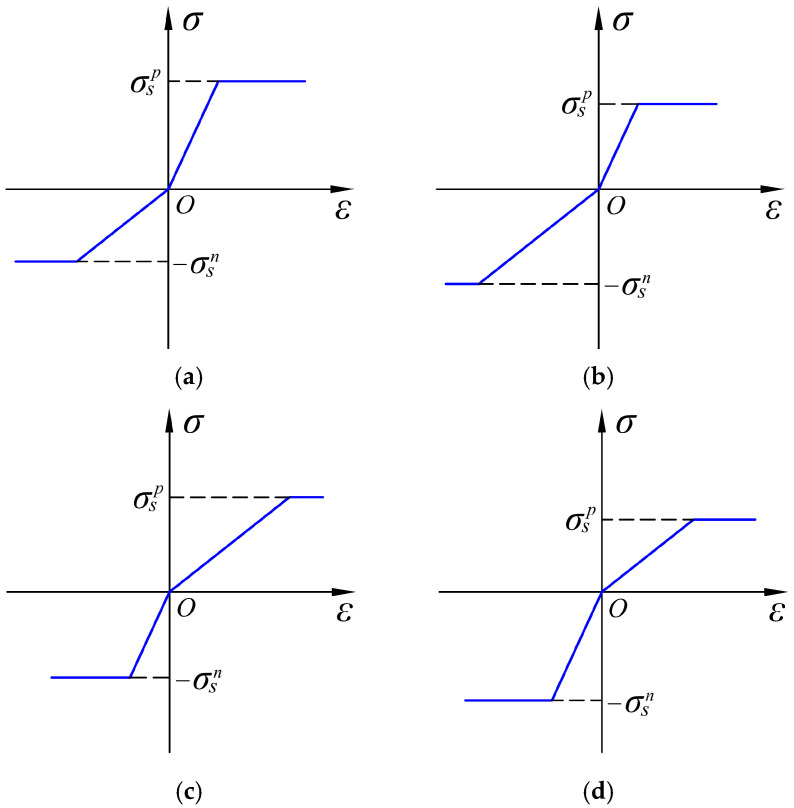
An elastic and ideal plastic model with bimodular and SD effects, where (**a**) *E_p_* > *E_n_*, σ*_s_^p^* > σ*_s_^n^*; (**b**) *E_p_* > *E_n_*, σ*_s_^p^* < σ*_s_^n^*; (**c**) *E_p_* < *E_n_*, σ*_s_^p^* > σ*_s_^n^*; (**d**) *E_p_* < *E_n_*, σ*_s_^p^* < σ*_s_^n^*.

For any material, it is very important to establish the stress–strain relationship in the one-dimensional case, because it is an important basis for modeling materials under complex stress regimes. If we consider all the cases that may contain both bimodular and SD effects, there will be a total of four cases, see Figure 1, in which *E_p_* and *E_n_* are the tensile elastic modulus and compressive one, respectively, and σ*_s_^p^* and σ*_s_^n^* are the yield strength in tension and compressive, respectively. It is easily observed from Figure 1 that the change in trend of elastic modulus and yield strength is inconsistent, which means when the tensile modulus is bigger than the compressive one, the tensile yield strength may be less than the compressive. This phenomenon directly indicates that for a pure bending beam, the tensile edge and the compression edge do not yield simultaneously. Obviously, the existence of four scenarios has further complicated the analysis when tension–compression asymmetry of materials is further used for the structural analysis.

Cantilever beams, as a basic structural form, have significant applications in multiple fields, ranging from mechanical engineering to microelectromechanical systems and even to biomedicine and other areas. Cantilever structures are playing an irreplaceable role. Meanwhile, the cantilever beam is a beam-type structure where one end is fixed and another is free. This structural form results in a large deflection at the free end and a concentrated bending moment at the fixed end. Therefore, in the analysis and design, special attention needs to be paid. At the same time, the cantilever beam is a statically determinate structure. Under the action of large loads, once a plastic hinge forms at the fixed end, the cantilever beam will lose its load-bearing capacity, which also needs to be specially considered in the design. Thus, for the optimization design and refined analysis of cantilever beams, taking into full account the mechanical properties of the materials will provide valuable references for related design. For instance, what would happen if we consider the material’s tension–compression asymmetry? According to the existing literature on elastic–plastic analysis and rigid-plastic analysis of beams [41,42,43,44,45,46,47,48], there are few works that consider both bimodular effects and SD effects.

Two important materials in civil engineering which are common to us, concrete and steel, also show obvious antisymmetric characteristics of tension and compression. Concrete, as one of the most common construction materials, has a compressive strength that is much higher than its tensile strength. For steel, whether the compressive strength and tensile strength are identical depends mainly on the carbon content as well as its plasticity and brittleness. In the plastic limit analysis of structures, the difference in tensile and compressive strength of the materials will greatly affect the final plastic ultimate load and deformation. Therefore, this work has certain practical significance for the actual structure in civil engineering.

This study is an important extension of our previous study [49], in which we have established the basic mechanical model for a pure bending beam with tension–compression asymmetry, as shown in Figure 1. In this work, we will investigate the elastic–plastic deformation of cantilever beams with tension–compression asymmetry theoretically. The structure of this paper is as follows. In Section 2, we will first present the mechanical model and basic assumption and then derive the elastic–plastic deflection solutions in loading and unloading. Section 3 is the FEM simulation and comparison with analytical solutions. Section 4 is the parameter study, in which the influence of two important parameters, modulus ratio and yield strength ratio in tension and compression, on the elastic–plastic deformation are discussed. Section 5 is the concluding remarks.

## 2. Elastic–Plastic Analysis of Cantilever Beam with Tension–Compression Asymmetry

In this section, based on the elastic–plastic analysis, we will obtain the deflection solution of cantilever beams with tension–compression asymmetry in loading and unloading.

### 2.1. Mechanical Model and Basic Assumptions

**Figure 2:**
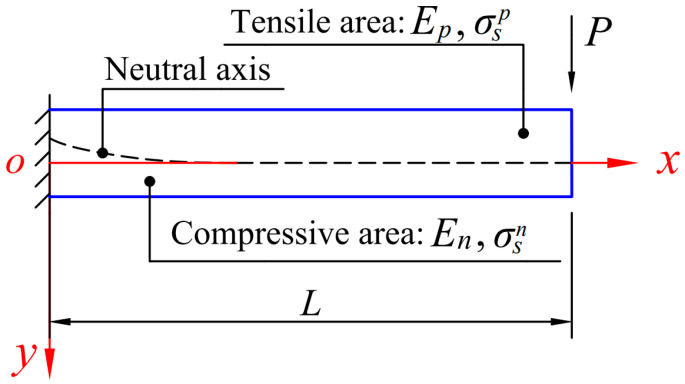
Schematic diagram of a cantilever beam with tension–compression asymmetry under the concentrated force at the free end.

Here we consider a cantilever beam with tension–compression asymmetry and under the action of a concentrated force *P* acting at the free end, as shown in Figure 2, in which the length of the beam is *L*, the *x*-axis is placed on the neutral axis, and the *y*-axis is set vertically downward. Note that under the action of concentrated force *P*, the beam will bend and form two different states of force application, that is, above the neutral axis, the beam section is tensile, and thus the corresponding elastic modulus and yield strength will be *E_p_* and σ*_s_^p^*, and below the neutral axis, the beam section is compressive, and thus the corresponding elastic modulus and yield strength will be *E_n_* and σ*_s_^n^*. Unlike the classical analysis, here, due to the elastic bimodular effect, the neutral axis does not coincide with the geometric centerline of the beam, as shown in Figure 2. In addition, due to different yield strengths in tension and compression, the yielding will begin at the fixed end and then extend from left to right, which is also asymmetrical in terms of its up-down orientation. This fact forces us to distinguish between the two situations where the tensile edge yields first or the compressive edge yields first, thereby further complicating the elastic–plastic response of the cantilever beam. For the convenience of the next analysis, we define two important ratios as

(1) $$a=\frac{E_{p}}{E_{n}},\;\;\;\;\;c=\frac{{\sigma_{S}}^{p}}{{\sigma_{S}}^{n}},$$

which stand for the relative magnitude of the tension–compression modulus and the yield strength. Also note that due to the shallow beam, the plane section assumption holds and the influences of shear stress on the position of the neutral axis and deformation are negligible, which helps to simplify the analysis.

### 2.2. Elastic–Plastic Analysis in Loading

Via the equilibrium conditions, the bending moment of the beam is

(2) M(x)=(L−x)P.

It is obvious that the maximum bending moment takes place at the fixed end, which gives, at *x* = 0,

(3) M=LP.

When the *P* increases up to

(4) $$P\to P_{e}=\frac{M_{e}}{L},$$

the outermost fiber of section *x* = 0 begins to yield, and thus *M_e_* is called as the elastic limit bending moment which has been determined in our previous study [49], and *P_e_* is called as the elastic limit load, and

(5) $$P_{e}=\frac{M_{e}}{L}=\left\{\begin{array}{l} \frac{bh^{2}\sqrt{E_{n}}{\sigma_{S}}^{p}}{3(\sqrt{E_{p}}+\sqrt{E_{n}})L},\;\;\;\;\;\;\;\;\sqrt{a}\geq c\\ \frac{bh^{2}\sqrt{E_{p}}{\sigma_{S}}^{n}}{3(\sqrt{E_{p}}+\sqrt{E_{n}})L},\;\;\;\;\;\;\;\;\sqrt{a}\leq c \end{array}\right.,$$

in which two different cases are considered and, when *a*^1/2^ ≥ *c*, the tensile edge yields first; when *a*^1/2^ ≤ *c*, the compressive edge yields first.

**Figure 3:**
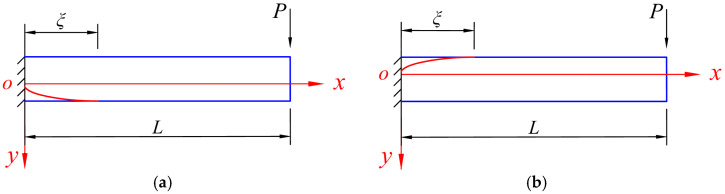
The development of the plastic zone of the beam when *P* > *P_e_*, in which (**a**) the compressive edge yields first and (**b**) the tensile edge yields first.

When *P* > *P_e_*, the bending moment still follows Equation (2), while at the same time, part of the beam has already entered an elastic–plastic state, and the development of the plastic zone can be shown in Figure 3, which distinguishes whether the tensile edge yields first or the compressive edge yields first. Let the cross-section that just begins to enter the plastic state be at *x* = *ξ*, we have

(6) $$M=(L-\xi )P=M_{e}.$$

Note that this is only the elastic–plastic stage I, that is, either the tensile edge or the compressive edge yields first, but the two edges do not both yield, which differentiates this stage from the next elastic–plastic stage II.

When the bending moment at the fixed end, *M*(0) = *M*_12_, the load *P* becomes

(7) $$P\to P_{12}=\frac{M_{12}}{L},$$

in which, *M*_12_ stands for the bending moment at which the section starts to enter the elastic–plastic stage II, and *M*_12_ has been determined in our previous study [49].

**Figure 4:**
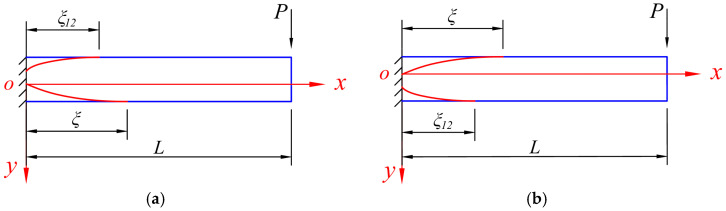
The development of the plastic zone of the beam when *P* > *P*_12_, in which (**a**) the compressive edge yields first and (**b**) the tensile edge yields first.

Further, when *P* > *P*_12_, the bending moment still follows Equation (2), while at the same time, the beam enters the elastic–plastic stage II, and the development of the plastic zone can be shown in Figure 4, which still distinguishes whether the tensile edge yields first or the compressive edge yields first. Let the cross-section that just starts to enter the elastic–plastic stage II be at *x* = *ξ*_12_, we have

(8) $$M=(L-\xi_{12})P=M_{12}.$$

When *M*(0) = *M_S_*, the section at the fixed end *x* = 0 achieves the plastic limit state; in this case, the plastic limit load *P_S_* may be determined by the corresponding plastic limit bending moment *M_S_*, which has been derived in [49]:

(9) $$M_{S}=\frac{bh^{2}{\sigma_{S}}^{p}{\sigma_{S}}^{n}}{2({\sigma_{S}}^{p}+{\sigma_{S}}^{n})}.$$

Combining Equations (1) and (5), we may derive the plastic limit load:

(10) $$P_{S}=\frac{M_{S}}{L}=\frac{bh^{2}{\sigma_{S}}^{p}{\sigma_{S}}^{n}}{2({\sigma_{S}}^{p}+{\sigma_{S}}^{n})L}=\left\{\begin{array}{l} \frac{3}{2}\frac{\sqrt{a}+1}{c+1}P_{e},\;\;\;\;\;\;\;\;\;\;\;\;\sqrt{a}>c\\ \frac{3}{2}\frac{\sqrt{1/a}+1}{1/c+1}P_{e},\;\;\;\;\;\;\;\;\sqrt{a}<c \end{array}\right..$$

**Figure 5:**
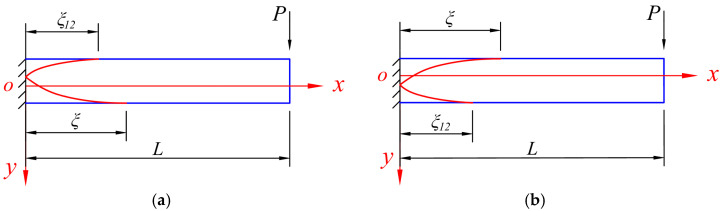
The development of the plastic zone of the beam when *P* = *P*_S_, in which (**a**) the compressive edge yields first and (**b**) the tensile edge yields first.

At this time, the entire section at the fixed end enters a plastic flow state and further loses its load-bearing capacity, and the development of the plastic zone can be shown in Figure 5, in which at the fixed end, the intersection point of the two curves is where the plastic hinge occurs.

The *ξ* corresponding to *P_S_* may be determined via Equations (6) and (10),

(11) $$\xi (P=P_{S})=\left\{\begin{array}{l} \left(1-\frac{2}{3}\cdot \frac{1+c}{1+\sqrt{a}}\right)L,\;\;\;\;\;\;\;\;\;\;\;\;\sqrt{a}\geq c\\ \left(1-\frac{2}{3}\cdot \frac{1+1/c}{1+\sqrt{1/a}}\right)L,\;\;\;\;\;\;\;\;\sqrt{a}\leq c \end{array}\right..$$

If *a*^1/2^ = *c*, we may have *ξ*(*P* = *P*_S_) = *L*/3; this value is exactly the familiar result of the cantilever beam without tension–compression asymmetry.

If the bending moment at a certain section of the beam reaches the plastic limit bending moment, a plastic hinge will generate. In this case, the corresponding curvature can increase arbitrarily, but the bending moment the section bears will remain unchanged at the plastic limit bending moment. For the cantilever beam studied in this work, the structure will be damaged when a plastic hinge appears. Therefore, the load range within which this structure can serve normally is *P* < *P*_S_.

### 2.3. Deflection of the Beam in Loading

Next, we will analyze, in loading, the deflection of the cantilever beam with the tension–compression asymmetry.

#### 2.3.1. Deflection in Elastic Stage

When *P* < *P_e_*, the whole beam is in the elastic stage, and the relationship of curvature and bending moment of the beam may be expressed as

(12) $$\frac{K(x)}{K_{e}}=\frac{M(x)}{M_{e}},$$

in which *K_e_* is the elastic limit curvature. Combining Equations (2), (4), and (12), we have

(13) $$\frac{d^{2}w}{dx^{2}}=\left(1-\frac{x}{L}\right)\left(\frac{P}{P_{e}}\right)K_{e}.$$

Considering the boundary conditions of the fixed end,

(14) $$w(0)=\frac{dw(0)}{dx}=0,$$

we have

(15) $$w(x)=\left(\frac{x^{2}}{2}-\frac{x^{3}}{6L}\right)\left(\frac{P}{P_{e}}\right)K_{e}.$$

In particular, when *P* = *P_e_*, the deflection at the free end of the beam, *δ_e_*, is

(16) $$\delta_{e}=w(L)=\frac{L^{2}}{3}K_{e}.$$

It is found that the forms of Equations (12)–(16) are consistent with the analytical results of beams having the same mechanical properties in tension and compression, with the only difference in the specific expressions of *P_e_*, *M_e_*, and *K_e_*.

#### 2.3.2. Deflection in Elastic–Plastic Stage

**Figure 6:**
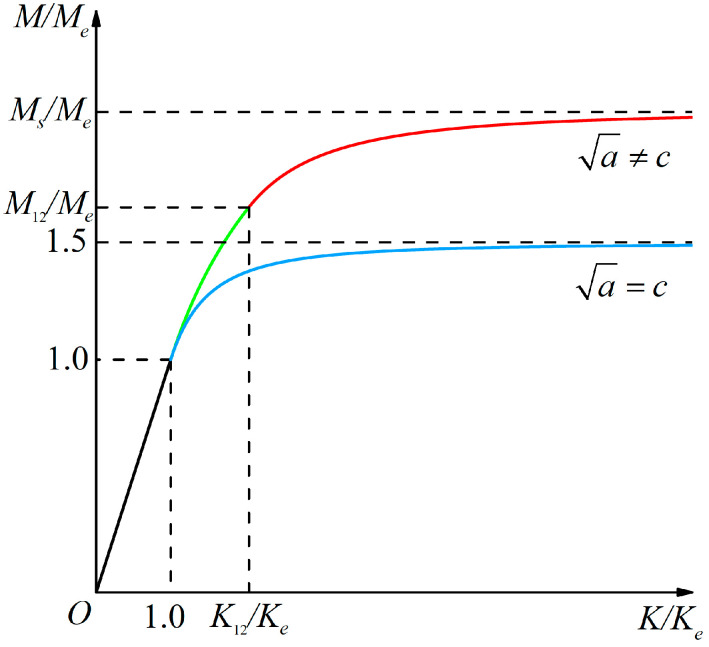
The curves of moment–curvature relationship of bending beams with tension–compression asymmetry.

When *P_e_* < *P* < *P_S_*, the beam is in the elastic–plastic stage. The relationship curves of bending moment *M* and curvature *K* of the beam have been plotted in our previous study [49], see Figure 6, in which *M_e_*, *M*_12_, and *M_S_* are the elastic limit bending moment, the bending moment from the elastic–plastic stage I to elastic–plastic stage II, and the plastic limit bending moment, respectively; *K_e_* and *K*_12_ are the corresponding curvatures in these stages. Figure 6 clearly shows the elastic stage (*M*/*M_e_* < 1), elastic–plastic stage I (1 < *M*/*M_e_* < *M*_12_/*M_e_*), and the elastic–plastic stage II (*M*_12_/*M_e_* < *M*/*M_e_* < *M_S_*/*M_e_*) up to the plastic limit stage (*M_S_*/*M_e_*).

Note that in Figure 6, the curve for *a*^1/2^ = *c* represents the classical case without tension–compression asymmetry, and this curve may be expressed in such a function [50] as

(17) $$\frac{K}{K_{e}}=\frac{1}{\sqrt{3-2\frac{M}{M_{e}}}}.$$

For the curve of *a*^1/2^ ≠ *c* with tension–compression asymmetry, its function expression is hard to give due to the fact that the introduction of tension–compression asymmetry complicates the problem and that the whole elastic–plastic stage is divided into stage I and stage II. For this purpose, we need to find an appropriate function to simulate the curve of *a*^1/2^ ≠ *c*. Note that the two curves have the same developmental trend, both being continuous and smooth, and thus the function of curve *a*^1/2^ = *c* may be used for the reference of the taken function of curve *a*^1/2^ ≠ *c*. Finally, we take the following function for the curve *a*^1/2^ ≠ *c*

(18) $$\frac{K}{K_{e}}=\frac{1}{\sqrt{A-B\frac{M}{M_{e}}}}-C,$$

in which, *A*, *B*, and *C* are dimensionless undetermined coefficients which are introduced here to simulate the curve and may be determined via certain control conditions. For the determination of *A*, *B*, and *C*, we need to express the whole curve in the elastic stage and elastic–plastic stage as follows, not differentiating the elastic–plastic stage I from stage II,

(19) $$\left\{\begin{array}{l} K_{1}=\frac{M}{M_{e}}K_{e},\;\;\;\;\;\;\;\;\;\;\;\;\;\;\;\;\;\;\;\;\;\;\;\;\;\;\;\;\;\;\;\;\;\;\;\;\;\;\;\;\;\;M\leq M_{e}\\ K_{2}=\frac{K_{e}}{\sqrt{A-B\frac{M}{M_{e}}}}-CK_{e},\;\;\;\;\;\;\;\;\;\;M_{e}\leq M<M_{S} \end{array}\right.,$$

in which *K*_1_ and *K*_2_ are the curvature in the elastic stage and elastic–plastic stage, respectively.

First, the curve of *a*^1/2^ ≠ *c* is continuous and smooth, as observed in Figure 6, and thus we have

(20) $$K_{1}(M_{e})=K_{2}(M_{e}),\;\;\frac{dK_{1}(M_{e})}{dM}=\frac{dK_{2}(M_{e})}{dM}.$$

In addition, when the bending moment increases to its plastic limit, we have

(21) $$\underset{M\to M_{S}}{\lim}K_{2}=\infty ,$$

this gives

(22) $$A-B\frac{M_{S}}{M_{e}}=0.$$

By combining Equations (20) and (22) and also letting *m_s_* = *M_S_*/*M_e_*, we obtain

(23) $$A=\frac{m_{s}}{4{\left(m_{s}-1\right)}^{3}},\;\;B=\frac{1}{4{\left(m_{s}-1\right)}^{3}},\;\;C=2m_{s}-3.$$

Specifically, if *m_s_* = 3/2, which corresponds to the case without tension–compression asymmetry, we will have *A* = 3, *B* = 2, and *C* = 0, and Equation (18) may reduce into Equation (17); this fact verifies the correctness of the function form for curve *a*^1/2^ ≠ *c* from the side.

To solve the deflection further, we rewrite Equation (19) by considering Equations (2) and (4):

(24) $$\frac{d^{2}w}{dx^{2}}=\left\{\begin{array}{l} \frac{K_{e}}{\sqrt{A-B\left(1-\frac{x}{L}\right)\frac{P}{P_{e}}}}-CK_{e},\;\;\;\;\;\;\;\;\;\;0\leq x\leq \xi \\ \left(1-\frac{x}{L}\right)\left(\frac{P}{P_{e}}\right)K_{e},\;\;\;\;\;\;\;\;\;\;\;\;\;\;\;\;\;\;\;\;\;\;\;\;\;\xi \leq x\leq L \end{array}\right..$$

Note that the beam segment at 0 ≤ *x* ≤ *ξ* is in the elastic–plastic stage, while the beam segment at *ξ* ≤ *x* ≤ *L* is still in elastic stage. Integrating twice to *x*, we have

(25) $$\left\{\begin{array}{l} w_{1}=\frac{4K_{e}{P_{e}}^{2}L^{2}{\left[A-B\left(1-\frac{x}{L}\right)\frac{P}{P_{e}}\right]}^{3/2}}{3B^{2}P^{2}}-\frac{1}{2}CK_{e}x^{2}+Ex+F,\;\;\;\;\;\;0\leq x\leq \xi \\ w_{2}=\left(\frac{x^{2}}{2}-\frac{x^{3}}{6L}\right)\frac{P}{P_{e}}K_{e}+Gx+H,\;\;\;\;\;\;\;\;\;\;\;\;\;\;\;\;\;\;\;\;\;\;\;\;\;\;\;\;\;\;\;\;\;\;\;\;\;\;\;\;\;\;\;\;\;\;\;\;\xi \leq x\leq L \end{array}\right.,$$

in which *w*_1_ is the deflection of beam segment in the elastic–plastic stage, located near the fixed end; *w*_2_ is the deflection of beam segment in the elastic stage, located near the free end; and *A*, *B*, and *C* are known, while *E*, *F*, *G*, and *H* are undetermined coefficients which may be determined by the following boundary conditions and continuity conditions:

(26) $$\left\{\begin{array}{l} w_{1}(0)=0,\;\;\;\frac{dw_{1}(0)}{dx}=0,\\ w_{1}(\xi )=w_{2}(\xi ),\;\;\;\frac{dw_{1}(\xi )}{dx}=\frac{dw_{2}(\xi )}{dx}, \end{array}\right.$$

thus, *E*, *F*, *G*, and *H* are determined as

(27) $$\left\{\begin{array}{l} E=-\frac{2P_{e}LK_{e}\sqrt{A-B\frac{P}{P_{e}}}}{BP},\;\;F=-\frac{4{P_{e}}^{2}L^{2}K_{e}{\left(A-B\frac{P}{P_{e}}\right)}^{\frac{3}{2}}}{3B^{2}P^{2}},\\ G=-\frac{LK_{e}\left\{2{P_{e}}^{2}\left(\sqrt{A-B\frac{P}{P_{e}}}-\sqrt{A-B}\right)+B\left[\left(C+\frac{1}{2}\right)P_{e}+\frac{P}{2}\right](P-P_{e})\right\}}{BPP_{e}},\\ H=\frac{L^{2}K_{e}}{6B^{2}P^{2}P_{e}}\left\{\begin{array}{l} 4{P_{e}}^{2}\left[B(-3P+P_{e})+2AP_{e}\right]\sqrt{A-B}\\ +3B^{2}\left[\left(C+\frac{2}{3}\right)P_{e}+\frac{P}{3}\right]{\left(P-P_{e}\right)}^{2}-8{P_{e}}^{3}{\left(A-B\frac{P}{P_{e}}\right)}^{3/2} \end{array}\right\}. \end{array}\right.$$

The deflection at the free end (*x* = *L*) may be obtained as

(28) $$\delta =w_{2}(L)=\frac{K_{e}L^{2}}{6B^{2}P^{2}}\left\{\begin{array}{l} -4{P_{e}}^{2}\left(2A+B\frac{P}{P_{e}}\right)\sqrt{A-B\frac{P}{P_{e}}}\\ +4{P_{e}}^{2}(2A+B)\sqrt{A-B}+3B^{2}\left[\left(C+\frac{2}{3}\right){P_{e}}^{2}-CP^{2}\right] \end{array}\right\}.$$

Further, if letting *P* = *P_S_* in Equation (28), we may obtain the limit deflection at the free end:

(29) $$\delta_{S}=\frac{10{m_{s}}^{3}-15{m_{s}}^{2}+6m_{s}+1}{6{m_{s}}^{2}}K_{e}L^{2}.$$

In particular, if returning to the classical case without tension–compression asymmetry, that is, *E_p_* = *E_n_* = *E* and σ*_s_^p^* = σ*_s_^n^* = σ*_s_*, we have, in this case, *m_s_* = 3/2, and the limit deflection at *x* = *L* will become

(30) $$\delta_{S}=\frac{20}{27}K_{e}L^{2},$$

which is exactly the familiar result of the classical problem without tension–compression asymmetry [50]. Obviously, this consistency verifies again the correctness of our whole analysis from the side.

### 2.4. Deflection of the Beam in Unloading

When the beam is loaded up to *P* = *P*_0_ (*P*_0_ > *P_e_*), the deflection at *x* = *L* gives, according to Equation (28),

(31) $$\delta_{0}=w_{2}(L)=\frac{K_{e}L^{2}}{6B^{2}{P_{0}}^{2}}\left\{\begin{array}{l} -4{P_{e}}^{2}\left(2A+B\frac{P_{0}}{P_{e}}\right)\sqrt{A-B\frac{P_{0}}{P_{e}}}\\ +4{P_{e}}^{2}(2A+B)\sqrt{A-B}+3B^{2}\left[\left(C+\frac{2}{3}\right){P_{e}}^{2}-C{P_{0}}^{2}\right] \end{array}\right\}.$$

If now the beam will be unloaded, and during unloading the deformation of the beam conforms to the elastic law, from Equation (15) we may obtain the deflection change at *x* = *L* when the unloading amount is Δ*P* (Δ*P* < 0):

(32) $$\Delta \delta =\frac{K_{e}L^{2}\Delta P}{3P_{e}}.$$

Thus, after unloading, the residual deflection at the free end (*x* = *L*) will be

(33) $$\delta =\delta_{0}+\Delta \delta ==\frac{K_{e}L^{2}}{6B^{2}{P_{0}}^{2}}\left\{\begin{array}{l} -4{P_{e}}^{2}\left(2A+B\frac{P_{0}}{P_{e}}\right)\sqrt{A-B\frac{P_{0}}{P_{e}}}\\ +4{P_{e}}^{2}(2A+B)\sqrt{A-B}+3B^{2}\left[\left(C+\frac{2}{3}\right){P_{e}}^{2}-C{P_{0}}^{2}\right] \end{array}\right\}+\frac{K_{e}L^{2}\Delta P}{3P_{e}}.$$

Further, we consider the beam is first loaded up to *P_S_*, and then it is unloaded to zero. In this case, by substituting *P*_0_ = *P_S_* and Δ*P* = −*P_S_* into Equation (33), the residual deflection at the free end will be

(34) $$\delta_{s}^{0}=\frac{8{m_{s}}^{3}-15{m_{s}}^{2}+6m_{s}+1}{6{m_{s}}^{2}}K_{e}L^{2}.$$

For the beam without tension–compression asymmetry, that is, the classical beam problem, *m_s_* = 3/2. At this time, the residual deflection at the free end of the beam gives

(35) $$\delta_{s}^{0}=\frac{13}{54}K_{e}L^{2},$$

this is exactly the result of the classical problem [50]. Obviously, this consistency of the deflection in unloading verifies the correctness of the derivation once again.

## 3. Numerical Simulation and Comparison

In this section, we will conduct FEM simulation and compare the numerical results with the analytical solutions in Section 2.

ABAQUS 6.14.4 is adopted to conduct the numerical simulation. Due to the fact that there is no a different tensile-compressive materials model in ABAQUS, the user-defined material subroutine UMAT is resorted to. In our previous study [49], the program flow chart of the FEM simulation of the elastic–plastic analysis of bending beams and the corresponding UMAT subroutine were given in detail, and thus they are not provided here for the reason of paper length.

**Figure 7:**
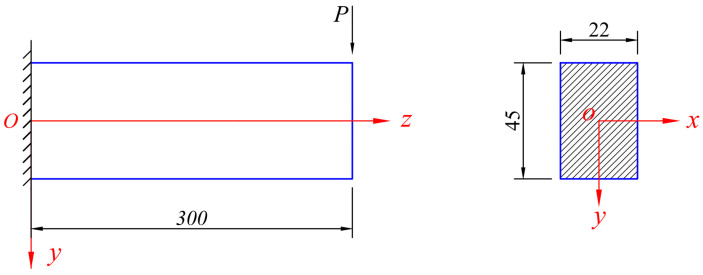
The structural model of cantilever beam in numerical simulation (unit: mm).

**Figure 8:**
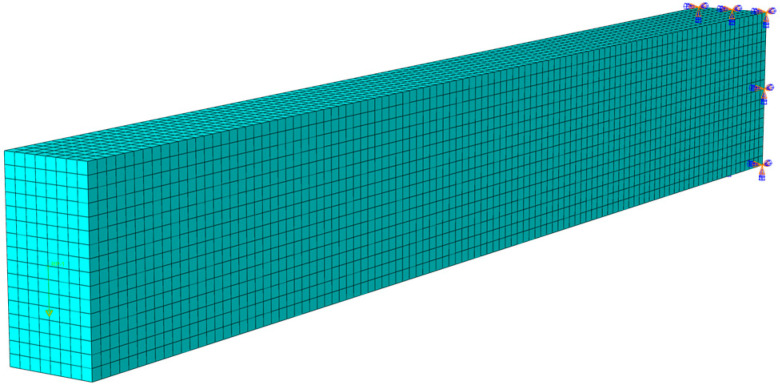
The solid model in numerical simulation.

The cantilever beam in our numerical simulation is shown in Figure 7, whose coordinate system *o-xyz* is established as follows: *x*-axis corresponds to the direction of beam thickness, *y*-axis to the height direction, and *z*-axis to the length direction. In the FEM simulation, we use the ABAQUS default convergence criterion with automatic time increment control and adopt the element C3D8 for the simulation. During the gridding, we set the global approximate size of the grid cells as 0.00285 and set the minimum size as 0.1 of the global size, thus generating a total of 8 × 16 × 105 = 13,440 cells, see Figure 8, and also including the fixed boundary constraints. The material parameters of the cantilever beam with tension–compression asymmetry are shown in Table 1.

In addition, it is usually assumed that the material follows the ideal elastic–plastic model and the associated flow law. For the cantilever beam with a concentrated force acting at the free end, we need to consider the influences of the bending stress *σ*_33_ and shear stress *τ*_23_, in which the subscripts 1, 2, and 3 correspond to *x*, *y*, and *z* direction of the beam, respectively, see Figure 7. Since the problem may be attributed to a plane stress problem, the yield function can be simply expressed as

(36) $$f=\left\{\begin{array}{l} \frac{1}{2}\sigma_{33}+\sqrt{\frac{1}{4}{\sigma_{33}}^{2}+{\tau_{23}}^{2}}-{\sigma_{S}}^{p},\;\;\;\;\;\;\;\;\;\;\;\;\sigma_{33}\geq 0\\ -\frac{1}{2}\sigma_{33}+\sqrt{\frac{1}{4}{\sigma_{33}}^{2}+{\tau_{23}}^{2}}-{\sigma_{S}}^{n},\;\;\;\;\;\;\;\;\;\sigma_{33}<0 \end{array}\right..$$

Finally, the numerical results under different concentrated forces are listed in Table 2, in which the analytical solution is obtained from Equation (28). Note that when using Equation (28) to compute the end displacement of the beam, we need to determine the following physical quantities: *A*, *B*, *C*, *P_e_*, *K_e_*, *L*, and *P*, in which *L* is the beam length, 300 mm, as shown in Figure 7 and *P* is the concentrated forces whose data are shown in Table 1, but *A*, *B*, *C*, *P_e_*, and *K_e_* in Equation (28) are still unknown for the moment. For this purpose, the first step is to judge whether the tensile edge yields first or the compressive edge yields first, thus determining these quantities. According to our previous study [49], when *a*^1/2^ ≥ *c*, the tensile edge yields first. It is easily obtained from Table 1 that here the tensile edge yields first. Thus from Equation (7a) in [49], we may have the elastic limit bending moment *M_e_* and elastic limit curvature *K_e_*:

(37) $$\left\{\begin{array}{l} M_{e}=\frac{bh^{2}\sqrt{E_{n}}{\sigma_{S}}^{p}}{3(\sqrt{E_{p}}+\sqrt{E_{n}})}\\ K_{e}=\frac{(\sqrt{E_{p}E_{n}}+E_{n}){\sigma_{S}}^{p}}{E_{p}E_{n}h} \end{array}\right..$$

Next, via the known *M_e_*, we may have the elastic limit load *P_e_* = *M_e_*/*L*; via the plastic limit bending moment *M_S_* shown in Equation (9), we also have the ratio *m_s_* = *M_S_*/*M_e_*; via Equation (23), the *A*, *B*, and *C* may be further obtained. Finally, via Equation (28), all end displacement values under different concentrated forces are computed, as shown in Table 2.

**Figure 9:**
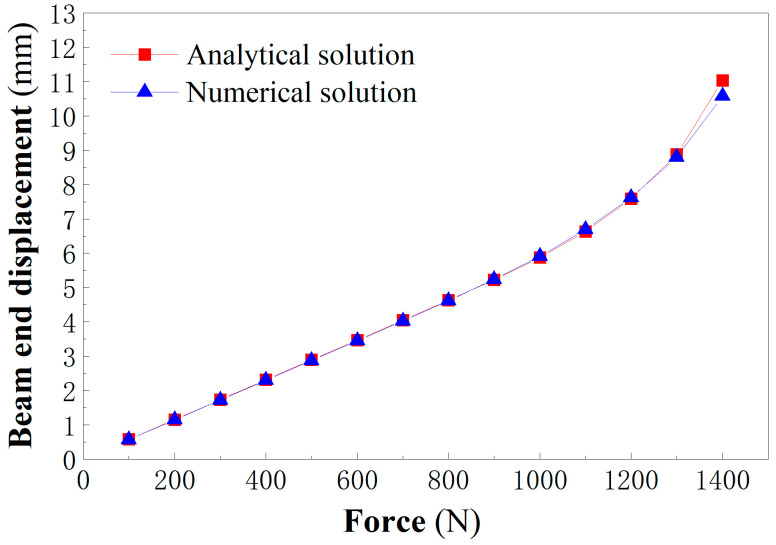
Comparison of analytical solution and numerical result for beam end displacement.

Figure 9 shows the comparison results of the analytical solutions and numerical solutions. It is readily found that they are very close, with slightly greater deviations under the greater loads; under the force *P* = 1400 N, the analytical solution is greater than the numerical result. In this case, the analytical solution gives 11.029 mm while the numerical result gives 10.579 mm, the relative error achieving 4.254%. This is because at this point the beam has approached the plastic limit state. In the approximate curvature function used in theoretical analysis, the bending moment of the section will approach the limit bending moment only when the section curvature *K* is positive infinity. This leads to a greater curvature of the beam and a greater displacement in the analytical solution. However, from a safety perspective, the theoretical solution is relatively conservative when approaching the yield limit. On the whole, the good agreement between the analytical solutions and the numerical results indicates that the assumptions adopted in the theoretical analysis is reasonable.

**Figure 10:**
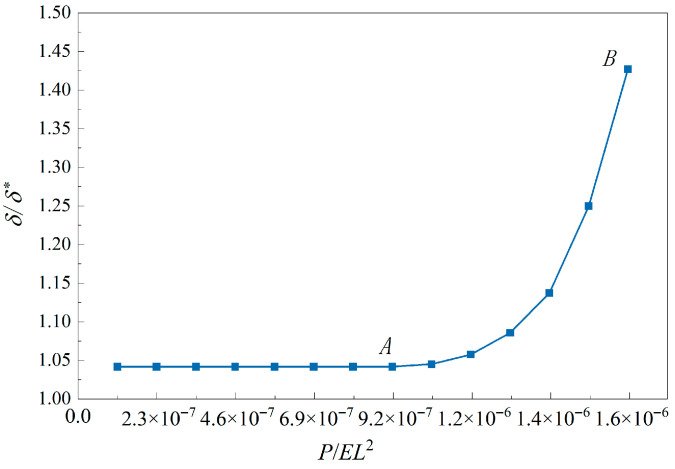
Normalized curve of end displacement vs. load, in which point *A* represents the beginning of elastic–plastic stage and point *B* represents the plastic limit state.

It is also interesting to directly compare the influence of tension–compression asymmetry independent of absolute scales. For this purpose, we normalize the analytical solution in Figure 9 and plot the normalized curve of end displacement vs. load, as shown in Figure 10, in which the concentrated force *P* is normalized as *P*/*EL*^2^ and end displacement *δ* as *δ*/δ*, and where *E* is the average value of *E_p_* and *E_n_* shown in Table 1, *L* is the beam length, δ* is the end displacement of a symmetric material model, and in computation the yield strength is taken as the average value of the tensile strength and compressive one.

It is easy to observe from Figure 10 that the curve first shows a horizontal state; after passing through point *A*, it shows a sharp upward trend until point *B*. Point *A* represents the beginning of elastic–plastic stage, which approximately occurs at load 800 N and corresponds to the value *δ*/δ* = 1.042, indicating that from 100 N to 800 N, the end displacement ratio keeps unchanged, and with the increase in load, the two displacements increase in equal proportion, so the ratio does not change. Point *B* represents the plastic limit state which approximately occurs at load 1400 N and corresponds to the value *δ*/δ* = 1.427, indicating that from 800 N to 1400 N, the increase in δ is faster than that of δ*. Starting from 1.042 and rising sharply to 1.427, it is proved that tension–compression asymmetry will enlarge the deformation response, especially when approaching the plastic limit state.

## 4. Results and Discussion

Since the validity of the analytical solution has been verified, in this section we will apply this solution to carry out the parameter study. In our study, there are two important parameters, *a* (= *E_p_*/*E_n_*) and *c* (= σ*_s_^p^*/σ*_s_^n^*), which greatly influence the elastic–plastic deformation of the cantilever beam.

First we note that in the elastic–plastic deformation process, the parameter *ξ* corresponding to the plastic limit load *P_S_* is an important quantity in the design since the *ξ* indicates when the plastic limit is achieved, that is, when the whole section at the fixed end of the cantilever beam has entered into the plastic flow state and further lost the ability to bear load, what is the distance to the fixed end? In the classical problem without the tension–compression asymmetry, this value gives *ξ*/*L* = 1/3. Here, if we consider the tension–compression asymmetry, is this value increasing or decreasing? For this purpose, we simply rewrite Equation (11) as

(38) $$\frac{\xi }{L}=\left\{\begin{array}{l} 1-\frac{2}{3}\cdot \frac{1+c}{1+\sqrt{a}},\;\;\;\;\;\;\;\;\;\;\;\;\;\;\;\sqrt{a}\geq c\\ 1-\frac{2}{3}\cdot \frac{1+1/c}{1+\sqrt{1/a}},\;\;\;\;\;\;\;\;\;\;\;\sqrt{a}\leq c \end{array}\right.,$$

which gives the change in ξ/*L* with *c* and *a* in an explicit form, in which *a*^1/2^ ≥ *c* corresponds to the tensile edge yields first, while *a*^1/2^ ≤ *c* corresponds to the compressive edge yield first, as indicated above.

**Figure 11:**
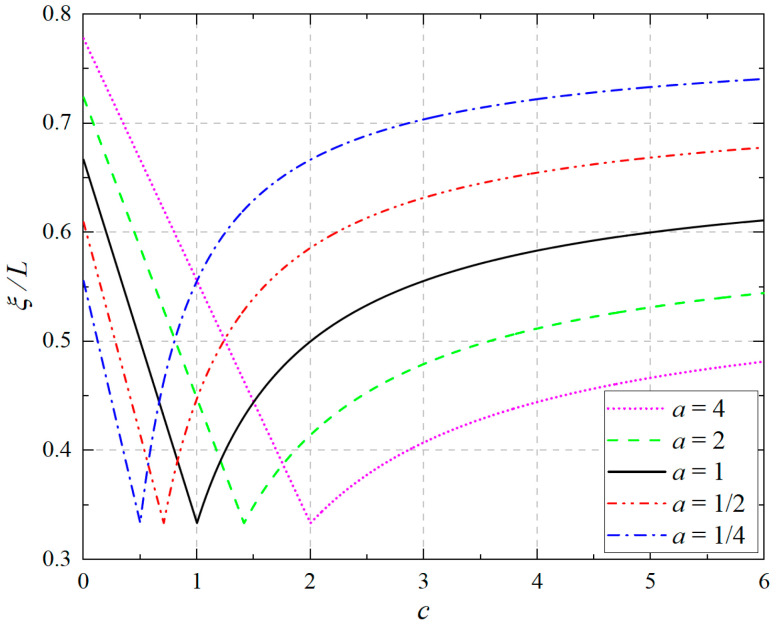
Variation in plastic zone length *ξ*/*L* with *c* under different *a*, in which *a* = *E_p_*/*E_n_*, *c* = σ*_s_^p^*/σ*_s_^n^*.

Figure 11 shows the change in *ξ*/*L* with *c* under varying *a* values. From Figure 11, it is observed that the five cases of *a* values all present a linear variation when *a*^1/2^ ≥ *c* and a curvilinear variation when *a*^1/2^ ≤ *c*, with the sharp point (*ξ*/*L* = 1/3) corresponding to *a*^1/2^ = *c*. Obviously, if the tensile edge yields first, the change in ξ/*L* with *c* is quick; compared to when the compressive edge yields first, the change is slow. This phenomenon indicates that when the tensile edge yields first, *ξ*/*L* is sensitive to *c*; when the compressive edge yields first and the *c* value is bigger, this is not the case.

For investigating the effect of two parameters *a* and *c* on the end displacement of the beam, we rewrite Equation (28) in a dimensionless form as follows, also considering Equation (16):

(39) $$\frac{\delta }{\delta_{e}}=\frac{1}{2B^{2}}\cdot \frac{{P_{e}}^{2}}{P^{2}}\left\{\begin{array}{l} -4\left(2A+B\frac{P}{P_{e}}\right)\sqrt{A-B\frac{P}{P_{e}}}\\ +4(2A+B)\sqrt{A-B}+3B^{2}\left[\left(C+\frac{2}{3}\right)-C\frac{P^{2}}{{P_{e}}^{2}}\right] \end{array}\right\},$$

in which, *A*, *B*, and *C* are dimensionless quantities themselves which have been given in Equation (23), and they all depend on the ratio *m_s_* = *M_S_*/*M*.

**Figure 12:**
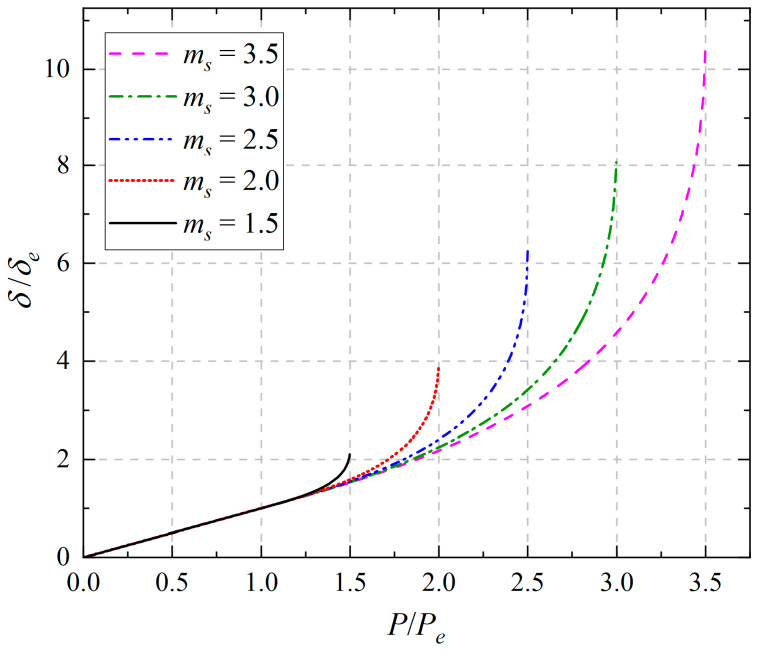
Relationship curves of end displacements and loads under different *m_s_* = *M_S_*/*M_e_*.

Figure 12 shows, under the different *m_s_* values, the relationship curves of end displacement (*δ*/*δ_e_*) and external load (*P*/*P_e_*), in which *m_s_* = 1.5 corresponds to the case without tension–compression asymmetry, while *m_s_* = 2.0, 2.5, 3.0, and 3.5 correspond to the other cases with tension–compression asymmetry. From Figure 12, it is easily observed that with the increase of *m_s_*, the proportion of the plastic displacement part will increase, and also the end displacement will present the same trend. Due to the fact that *m_s_* depends on the two important parameters, *a* and *c*, different taken values of *m_s_* reflect the tension–compression asymmetry (different moduli effect in elasticity and SD effect in plasticity). Finally, it can be concluded that the consideration for the tension–compression asymmetry will enlarge the influence on the displacement during the elastic–plastic mechanical response.

Similarly, combining Equations (29) and (16), we may have the dimensionless form of the ultimate deflection at the free end:

(40) $$\frac{\delta_{S}}{\delta_{e}}=\frac{10{m_{s}}^{3}-15{m_{s}}^{2}+6m_{s}+1}{2{m_{s}}^{2}},$$

which depends on the ratio *m_s_*. Combining Equations (34) and (16), we may have the dimensionless residual deflection at the free end as follows when the beam is first loaded up to *P_S_* and then unloaded to zero:

(41) $$\frac{{\delta_{S}}^{0}}{\delta_{e}}=\frac{8{m_{s}}^{3}-15{m_{s}}^{2}+6m_{s}+1}{2{m_{s}}^{2}},$$

which also depends on the ratio *m_s_*.

**Figure 13:**
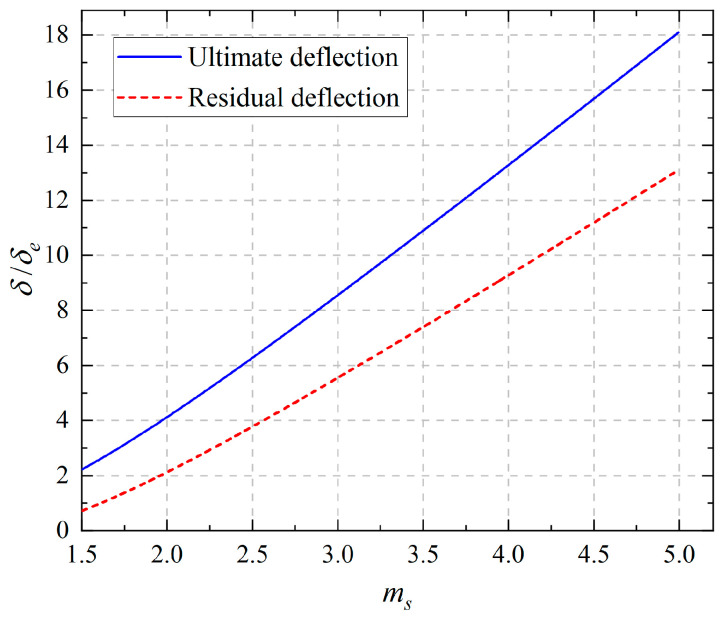
Relationship curves of ultimate deflection, residual flection, and *m_s_* = *M_S_*/*M_e_*.

Figure 13 shows the change in ultimate deflection (*δ_S_*/*δ_e_*) and the residual deflection (*δ_S_*^0^/*δ_e_*) at the free end with the increase of *m_s_*. From Figure 13, it is easily observed that the ultimate deflection at the free end is greater than the residual deflection; the two curves of ultimate deflection and residual deflection both have their minimum values at *m_s_* = 1.5, and they are *δ_S_*/*δ_e_* = 20/9 and *δ_S_*^0^/*δ_e_* = 13/18, which indicates the tension–compression asymmetry will enlarge the two deflections, and more importantly, the more obvious the tension–compression asymmetry is, the larger these two deflection values will be.

## 5. Concluding Remarks

In this work, we theoretically investigate the elastic–plastic deformation of a cantilever beam with a concentrated force acting on the free end. The material used to construct the cantilever beam exhibits tension–compression asymmetry, that is, in the elastic deformation stage, different elastic moduli in tension and compression are considered, while at the same time, in the plastic deformation stage, the strength differential effect is also considered. The numerical simulation further validates the theoretical solution. The following conclusions can be drawn.

1. When the fixed end of the cantilever beam forms a plastic hinge, the plastic zone length from the fixed end no longer keeps the classical value *ξ*/*L* = 1/3 and will increase if considering the tension–compression asymmetry.
2. The consideration for the tension–compression asymmetry will enlarge the influence on the displacement during the elastic–plastic mechanical response; the greater *m_s_* values will produce the greater end displacements. Similarly, the end displacement in the classical problem without tension–compression asymmetry is minimal.
3. The tension–compression asymmetry will enlarge the ultimate deflection and residual deflection of the free end of the beam, and more importantly, the more obvious the tension–compression asymmetry is, the larger these two deflection values will be.

In conclusion, the consideration for tension–compression asymmetry will enlarge the elastic–plastic deformation response of the cantilever beam, especially when approaching the plastic limit state, which should be given sufficient attention in the design of the plastic limit of structures, especially for the material which have obvious bimodular and SD effects. In addition, this study does not cover the issue of materials hardening when entering the plastic yielding, which can be considered in the future.

## Figures and Tables

**Table 1:** Material parameters of MSL82 graphite (MPa).

Material Parameters	*E_p_*	*E_n_*	σ*_s_^p^*	σ*_s_^n^*
	7460	11,930	27.90	68.41

**Table 2:** The end displacements of cantilever beam with tension–compression asymmetry.

Concentrated Forces (N)	Analytical Solutions (mm)	Numerical Results (mm)	Relative Errors (%)
100	0.579	0.576	0.521
200	1.158	1.151	0.608
300	1.737	1.727	0.579
400	2.316	2.302	0.608
500	2.895	2.878	0.591
600	3.474	3.453	0.608
700	4.053	4.030	0.571
800	4.632	4.617	0.325
900	5.227	5.233	0.115
1000	5.878	5.910	0.541
1100	6.638	6.689	0.762
1200	7.584	7.620	0.472
1300	8.879	8.796	0.944
1400	11.029	10.579	4.254

## Data Availability

The original contributions presented in this study are included in the article. Further inquiries can be directed to the corresponding author.
